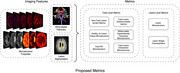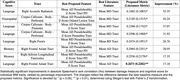# Quantifying the severity of white matter damage in Cerebral Small Vessel Disease

**DOI:** 10.1002/alz70861_108115

**Published:** 2025-12-23

**Authors:** Stylianos Charalampous, Frederik Barkhof, Carole H Sudre

**Affiliations:** ^1^ Hawkes Institute, University College London, London UK; ^2^ MRC Unit for Lifelong Health and Ageing, University College London, London UK; ^3^ Department of Radiology and Nuclear Medicine, Amsterdam UMC, Vrije Universiteit, Amsterdam Netherlands; ^4^ Queen Square Institute of Neurology and Centre for Medical Image Computing, University College London, London UK; ^5^ School of Biomedical Engineering and Imaging Sciences, King’s College London, London UK

## Abstract

**Background:**

Cerebral small vessel disease (CSVD) is a leading cause of cognitive impairment and dementia, with white matter hyperintensities (WMH) serving as a key neuroimaging marker. Traditional severity assessments using whole‐brain or tract‐wide averages often overlook lesion‐specific microstructural damage, limiting their sensitivity to cognitive outcomes. This study aimed to develop novel biomarkers integrating WMH‐focused metrics to improve the quantification of CSVD‐related damage.

**Method:**

A cross‐sectional analysis was conducted using multi‐modal data (T1‐weighted, T2‐FLAIR, and single‐shell diffusion‐weighted) from the Alzheimer’s Disease Neuroimaging Initiative (*n* =627; cognitively normal, n=361; mild cognitive impairment, n=196; Alzheimer’s disease, n=70). White matter pathways were reconstructed for 42 major tracts. Microstructural features of white matter change were characterized using Diffusion Tensor Imaging (DTI) and Neurite Orientation Dispersion and Density Imaging (NODDI) metrics extracted along these tracts.

A pseudo‐healthy reference group (WMH burden <1 ml, n=32) was used to construct Z‐score maps for each DTI and NODDI metric, quantifying deviations from normative white matter microstructure. Lesion‐specific metrics were computed by restricting DTI, NODDI, and Z‐score analyses to WMH, perilesional regions, and their union, and calculating a range of statistical measures.

Univariate Spearman correlations were used to assess associations between these metrics and cognitive composite scores (memory, language, executive, visuospatial). Each tract’s best‐performing conventional metric was compared to the best‐performing proposed metric, with statistical significance assessed using Steiger’s Z‐test.

**Result:**

The proposed metrics showed significant improvements in correlations with cognitive function, particularly in the visuospatial, language, and memory domains, when compared to literature‐based metrics. Incorporating Z‐score maps from pseudo‐healthy reference distributions consistently enhanced correlations relative to DTI and NODDI tract averages. Moreover, focusing the analysis on WMH and perilesional regions led to stronger associations with cognitive outcomes than tract‐wide approaches in several cases.

**Conclusion:**

Tract‐wide and lesion‐focused metrics derived from Z‐score maps relative to healthy reference distributions offer a more sensitive assessment of CSVD severity. Furthermore, measures restricted to WMH and their periphery outperform conventional tract‐wide approaches. These findings highlight the potential clinical utility of advanced microstructural imaging biomarkers for improved prediction of cognitive impairment and more precise evaluation of CSVD.